# Symbiont‐Mediated Metabolic Shift in the Sea Anemone *Anthopleura elegantissima*

**DOI:** 10.1111/mec.17722

**Published:** 2025-03-17

**Authors:** Tyler J. Carrier, Holland Elder, Jason Macrander, James L. Dimond, Brian L. Bingham, Adam M. Reitzel

**Affiliations:** ^1^ Department of Biological Sciences University of North Carolina at Charlotte Charlotte NC USA; ^2^ Center for Computational Intelligence to Predict Health and Environmental Risks University of North Carolina at Charlotte Charlotte NC USA; ^3^ Department of Integrative Biology Oregon State University Corvallis OR USA; ^4^ Australian Institute of Marine Science Townsville Australia; ^5^ Biology Department Florida Southern College Lakeland FL USA; ^6^ Shannon Point Marine Center Western Washington University Anacortes WA USA; ^7^ Department of Environmental Sciences Western Washington University Bellingham WA USA

**Keywords:** cnidarian, coral, metabolomics, microbiome, symbiodiniaceae, transcriptomics

## Abstract

Coral reefs and their photosynthetic algae form one of the most ecologically and economically impactful symbioses in the animal kingdom. The stability of this nutritional mutualism and this ecosystem is, however, at risk due to increasing sea surface temperatures that cause corals to expel their symbionts. Symbioses with these microeukaryotes have independently evolved multiple times, and non‐coral cnidarians (e.g., sea anemones) serve as a valuable and insightful comparative system due to their ease of husbandry in the laboratory and their ability to shuffle different strains of their photosymbionts to acclimate to thermal conditions. This breadth of symbiont shuffling is exemplified by the sea anemone *Anthopleura elegantissima* , which naturally occurs in symbiosis with the dinoflagellate *Breviolum muscatinei* (formerly *Symbiodinium*) or the chlorophyte *Elliptochloris marina* as well as being aposymbiotic. Here, we assembled a draft genome and used multi‐omics to characterise multiple physiological levels of each phenotype. We find that *A. elegantissima* has symbiont‐specific transcriptional and metabolomic signatures, but a similar bacterial community dominated by a single *Sphingomonas* species that is commonly found in the cnidarian microbiome. Symbiosis with either eukaryotic symbiont resulted in differential gene expression and metabolic abundance for diverse processes spanning metabolism and immunity to reproduction and development, with some of these processes being unique to either symbiont. The ability to culture *A. elegantissima* with its phylogenetically divergent photosymbionts and perform experimental manipulations makes *A. elegantissima* another tractable sea anemone system to decode the symbiotic conversations of coral reef ecosystems and aid in wider conservation efforts.

## 1 Introduction

Animals across the tree of life have established microbial symbioses that are of deep evolutionary origin (Bordenstein and Theis 2015; McFall‐Ngai et al. 2013; Zilber‐Rosenberg and Rosenberg 2008). One of the primary functions of and incentives to cooperate in these partnerships is establishing and maintaining a metabolic mutualism (Kiers et al. 2011; Leigh 2010). Animals are often limited in their biosynthetic capabilities and, as a result, rely on symbionts to supplement their diet with various nutrients. Aphids, for example, feed on plant sap that is low in essential amino acids and require the endosymbiont *Buchnera* spp. to offset this deficiency (Douglas 1998), while diverse marine invertebrates associate with bacteria that can convert reduced substrates into host biomass (Dubilier et al. 2008). The nutritional symbiosis with arguably the most widespread ecological and economic impact occurs between corals and their photosynthetic algae (Hoegh‐Guldberg et al. 2007; Hughes et al. 2003).

Coral reefs are biodiversity hotspots that are metabolically sustained by their symbiotic relationship with dinoflagellates of the family Symbiodiniaceae (Berkelmans and van Oppen 2006; LaJeunesse et al. 2018; Stat et al. 2008). These protists reside in vesicles within the coral's endodermal cells and typically supply the host with ≥ 90% of its total energy, while the host provides them with a sheltered environment and inorganic nutrients (Davy et al. 2012). Symbiodiniaceae are phylogenetically and functionally diverse, and some corals shuffle the composition of Symbiodiniaceae strains with which they associate in response to ambient and anthropogenic changes in the abiotic environment (Cunning et al. 2015). The ability to host and shuffle Symbiodiniaceae extends to other members of the class Anthozoa. The sea anemone *Exaiptasia diaphana*, for example, is a well‐established experimental system for understanding cnidarian symbioses. Many of the cellular and molecular insights of cnidarian symbioses would not have been possible without *E. diaphana* (Davy et al. 2012; Weis et al. 2008).

**FIGURE 1:**
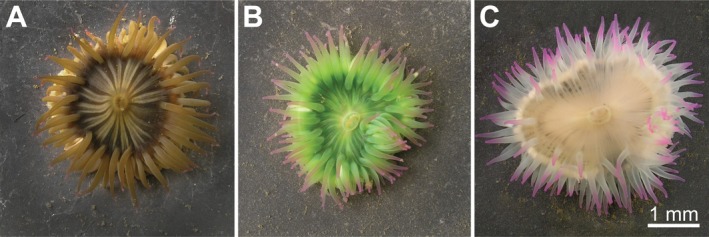
Symbiotic phenotypes of *Anthopleura elegantissima* . The sea anemone *A. elegantissima* occurs naturally in symbiosis with (A) the dinoflagellate *Breviolum muscatinei* (brown), (B) the chlorophyte *Elliptochloris marina* (green), or (C) without symbionts (white).

The sea anemone *Anthopleura elegantissima* is another long‐standing system that exemplifies the wide range of symbioses formed by cnidarians, which was first used to determine that symbionts transfer carbon to their cnidarian host (Muscatine and Hand 1958). This temperate, intertidal sea anemone naturally occurs in symbiosis with the dinoflagellate *Breviolum muscatinei* (formerly referred to as *Symbiodinium*), the chlorophyte *Elliptochloris marina* (which has only been reported in species of *Anthopleura*), or without symbionts (Figure 1; Lajeunesse and Trench 2000; Leutsch 2011; Muller‐Parker and Davy 2001). The symbionts that *A. elegantissima* associates with are primarily determined by latitude, with *E. marina* found along the upper latitudes and *B. muscatinei* observed in the lower latitudes of the geographic range for *A. elegantissima* (Secord and Augustine 2005). This biogeographic gradient coincides with temperature and irradiance across the Pacific Coast of North America, with *E. marina* preferring cooler, shaded habitats (Secord and Muller‐Parker 2005). This continental biogeographic signature can also be found along the vertical gradients of the intertidal zone; *A. elegantissima* is dominated by *B. muscatinei* high in the intertidal, *E. marina* low in the intertidal, and aposymbiotic individuals occur in dark crevices (Bergschneider and Muller‐Parker 2008; Dimond et al. 2011; Verde and McCloskey 2001, 2002).

These two symbionts exhibit strikingly different physiologies that directly contribute to the life history strategies and fitness of *A. elegantissima* . The chlorophyte *E. marina* lives at a 4 × higher density and grows 8 × faster than the dinoflagellate *B. muscatinei*, while each *B. muscatinei* cell is about 2.5 × more productive and can translocate 5 × more carbon to the host than an *E. marina* cell (Dimond et al. 2013; Verde and McCloskey 1996). Moreover, the metabolic products received by the sea anemone from these two symbionts are notably different: *E. marina* primarily translocates amino acids, while *B. muscatinei* translocates glycerol and sugars (Minnick 1984; Trench 1971a, 1971b); thus, these two symbionts serve different nutritional roles. As a result, these symbionts of *A. elegantissima* influence the host's balance between growth, asexual cloning, and sexual reproduction (Bergschneider and Muller‐Parker 2008; Bingham et al. 2014). Sea anemones hosting *E. marina* tend to reproduce sexually, while symbiosis with the more productive *B. muscatinei* promotes cloning by fission, a strategy that makes *A. elegantissima* a highly successful and spatially dominant member of the intertidal (Bingham et al. 2014).

Despite the long history of using *A. elegantissima* as a cnidarian system for symbiosis, we lack a modern molecular, metabolic, and microbial understanding of the partnership between *A. elegantissima* and its two phylogenetically distinct photosynthetic microeukaryotes. Therefore, the goal of this study was to characterise these symbiotic phenotypes under stable conditions in a laboratory‐based mesocosm. We did this by generating a reference genome and then comparing sea anemones of each symbiotic phenotype using transcriptomics, metabolomics, and amplicon‐based sequencing of the bacterial community to characterise multiple physiological levels. We find that the sea anemone *A. elegantissima* exhibits distinct transcriptional and metabolic profiles in each symbiotic phenotype, while the bacterial community remains unchanged. Furthermore, we observe that the bacterial community of *A. elegantissima* is dominated by a single *Sphingomonas* species, which is commonly found in the cnidarian microbiome.

## 2 Methods

### 2.1 Reference Genome for *A. elegantissima*

#### 2.1.1 Reference Genome: Identifying the Most Homozygous Individual

We generated a reference genome from the most homozygous individual within the geographic range of *A. elegantissima* . Individuals (*n* = 43 total, 3–4 individuals at each of the 11 sites) were collected throughout the latitudinal range (from northern Washington to southern California) of *A. elegantissima* (Figure S1). We then identified the individual with the lowest heterozygosity by multi‐locus single‐nucleotide polymorphism genotyping across the genome. Genomic DNA was extracted from three individuals from each site using the manufacturer's protocol in the E.Z.N.A. Tissue DNA Kit (Omega Bio‐tek; Norcross, GA, USA). Genomic regions were genotyped using type IIB restriction site‐associated DNA genotyping (i.e., 2b‐RAD) following the protocol from Wang et al. (2012). Libraries for all samples were sequenced in a single lane of 50 base pair single‐end reads on the Illumina 2500 platform at the Center for Genome Research and Biocomputing (CGRB; Oregon State University, OR, USA).

Raw reads were filtered using Trimmomatic (v. 0.39) to exclude those with low‐quality bases (i.e., a quality score of < 30) and any sequences containing Illumina adaptors (Bolger et al. 2014). A subset of the high‐quality, filtered reads was used to produce a *de novo* reference using Cluster Database at High Identity with Tolerance (CD‐HIT) (Fu et al. 2012). Reads from each individual were then mapped to the *de novo* reference, heterozygous loci were counted, and the ratio of heterozygous loci to all unique genotyped sites was calculated for each individual using custom scripts (see, https://github.com/Eli‐Meyer/2brad_utilities). The individual with the lowest heterozygous site frequency was then used for genome sequencing.

#### 2.1.2 Reference Genome: Assembly

We used DNA extracted from tentacle clips of the most homozygous sea anemone for genome sequencing. One paired‐end library and two mate‐pair libraries (one with a 4,000 base pair insert and another with a 6,000 base pair insert) were prepared for sequencing at the Center for Genome Research and Biocomputing (Oregon State University, OR, USA). A second paired‐end library was prepared and sequenced at the King Abdullah University of Science and Technology (Thuwal, Saudi Arabia). All short‐read libraries for genome assembly were sequenced on the Illumina HiSeq 3000 platform. Illumina adaptors were trimmed from all sequences, and low‐quality reads (i.e., quality scores < 20 at ≥ 15 bases) were removed using custom scripts (see, https://github.com/Eli‐Meyer/sequence_processing). Reads were then paired and interleaved—a process that matches each read with its reverse complement and writes them to a file so that they are in paired order in the sequence file for the assembler to read—using the reformat function of BBtool (Bushnell 2014). A draft genome using these short reads was assembled using AllPathsLGG (Table S2; Butler et al. 2008).

We improved this initial assembly using long‐read Oxford Nanopore sequences for *A. elegantissima* (Dimond et al. 2021). These published sequences were generated from an aposymbiotic individual using the MinION platform, which resulted in ~1.37 M reads that were between 5 and 12 kb (Table S2). The individuals used for short‐and long‐read sequencing were different and, thus, the standard process for genome polishing was not feasible. Therefore, we used the SSPACE‐long‐read assembler to polish this reference genome because it ignores differences in heterozygosity (Boetzer and Pirovano 2014). All contigs from the short‐read assembly were included, and all alignments of < 70 bp were removed.

Contaminant contigs from the eukaryotic photosymbiont and bacteria were subsequently removed from the polished *A. elegantissima* assembly using custom scripts (see, https://github.com/Eli‐Meyer/sequence_utilities). Specifically, a BLASTN search was implemented to compare the similarity of contigs in the *A. elegantissima* draft genome to that of *E. diaphana* , as well as the eukaryotic photosymbionts *B. minutum* and *S. microadriaticum* . Any putative *A. elegantissima* contig with a higher match score to the symbiont genomes than the *E. diaphana* genome was presumed to be symbiont DNA and consequently was removed from the final assembly. Contaminating bacterial sequences were also removed from the draft assembly using a custom script that implements BLASTN and compares the assembly to bacterial genomes in the National Center for Biotechnology Information (NCBI; see, https://github.com/Eli‐Meyer/sequence_utilities) (Table S2). Lastly, we manually removed contaminants and adaptors that were also identified by NCBI.

#### 2.1.3 Reference Genome: Annotation

We used MAKER to predict the protein sequences in the reference genome for *A. elegantissima* and then annotated those predicted proteins using Blast2GO (Cantarel et al. 2008; Conesa et al. 2005). Specifically, we used BLASTP against the Swiss‐Prot database within NCBI and provided Gene Ontology (GO) functional groups, with annotations being retained for proteins with a *p* value of < 0.001 (Table S3). We then mapped and annotated these BLAST results and merged the annotation of protein domains and families via InterProScan within Blast2GO (Table S3; Jones et al. 2014). Further classification of the metabolic functions was performed using the Kyoto Encyclopedia of Genes and Genomes (KEGG) within Blast2GO (Table S3; Kanehisa and Goto 2000).

### 2.2 Symbiont Phenotype: Sample Collection and Quantification

The sea anemone *A. elegantissima* was collected by hand from Swirl Island (WA, USA; 48.418333, 122.849444) in May 2013 (Figure 1). Sea anemones harbouring *B. muscatinei* or *E. marina* were collected from aggregations within 0.5 m of each other at the same tidal elevation. Aposymbiotic individuals were obtained from an adjacent shaded area under a large boulder, approximately 2 m from the symbiotic individuals. Sea anemones were brought back to the Shannon Point Marine Center (Anacortes, WA, USA) within an hour of collection and placed in an outdoor seawater table. Sea anemones were kept in individual glass dishes that were independently supplied with flowing seawater from multiple rows of tubing connected to a central manifold. The overflow water level in the seawater table was kept just below the level of the dishes and served to maintain the dishes at ambient seawater temperature, which averaged 10.5°C ± 1.0°C (mean ± S.D.) for the duration of the experiment. We chose to acclimate sea anemones in a common, moderate light environment, and, thus, we covered the seawater table with a layer of window screen to provide 45% ambient sunlight to reflect their habitat.

Following 20 days of acclimation, tissues (i.e., oral disc and tentacles; ~75 mg each) from all individuals (*n* = 6 per symbiont phenotype) were rinsed in deionised water before being preserved for transcriptomics in RNAlater, metabolomics in liquid nitrogen, and microbiome in ethanol. These samples were then shipped overnight on dry ice to the University of North Carolina at Charlotte. Additional tissues were stored at −80°C at the Shannon Point Marine Center to quantify symbiont abundance. This was performed by homogenising pieces of tentacles in 5‐μm filtered seawater using a small blender. Cell counts of symbionts in sea anemone homogenates were performed on a hemacytometer, with 4 replicate counts of 80–100 host cells. Protein content was then quantified on duplicate samples by mixing sea anemone homogenates with an alkaline copper solution and Folin–Ciocalteu reagent, which reacts with the copper‐protein complex to produce a colour peak at 750 nm. This peak was then measured with a spectrophotometer (Bausch & Limb Spectronic 20) and compared to a standard curve of protein concentrations created by a serial dilution of bovine serum albumin (Lowry et al. 1951). Symbiont counts were normalised to homogenate protein content to obtain symbiont density estimates, log_10_ transformed, and compared statistically using a one‐way analysis of variance (ANOVA).

### 2.3 Symbiont Phenotype: Host Transcriptomics

Total RNA was extracted from all samples using the RNeasy Plus Mini kit (Qiagen) according to the manufacturer's protocol. RNA yield and purity were quantified using a Nanodrop ND‐1000 spectrophotometer. Libraries were prepared with the Illumina Stranded mRNA Library kit and sequenced with an Illumina HiSeq (50 bp, paired‐end reads) by the Comprehensive Cancer Center at The Ohio State University (Columbus, OH, USA).

Raw reads were assessed for quality, trimmed using Trimmomatic (v. 0.39), and then mapped to the *A. elegantissima* genome using the STAR aligner (v. 2.7.9; Dobin et al. 2013) to account for spliced transcripts (Table S4). This count table was then imported into R (v. 4.4.0; Ihaka and Gentleman 1996), filtered of low abundance transcripts (i.e., mean count of less than 3), and normalised by the log‐transformed using the *rlog* function within DESeq2 (Love et al. 2014). The transcriptome was compared across symbiotic phenotype using the principal component analysis function “plotPCA,” visualised using ggplot (Wickham 2011), and compared statistically using an analysis of similarities (ANOSIM) in Vegan (Dixon 2003).

Differentially abundant transcripts were then identified by pairwise comparisons with DESeq2 and were visualised using a heatmap with pheatmap (Kolde 2019). Two numeric comparisons were performed to initially visualise these data. First, to inspect a broadscale pattern of symbiosis‐induced gene expression, average values for each differentially abundant transcript were made relative to that of aposymbiotic sea anemones. This resulted in expression patterns being either up or down regulated for both symbiont conditions—the latter being equivalent to up regulated for aposymbiotic sea anemones—or up regulated in one symbiont and down regulated in the other. Second, differentially abundant transcripts that also had a two‐fold difference in expression were compared with a Venn diagram (Oliveros 2015).

Gene ontology (GO) was assigned to all differentially abundant transcripts using the annotated genome for *A. elegantissima* . The three GO tables from each pairwise comparison were merged to identify four categories: (i) GO terms that were commonly found when associated with both symbionts, (ii and iii) GO terms that were unique to the association with *B. muscatinei* or *E. marina* , or (iv) GO terms that were unique to aposymbiotic individuals. GO terms for each category were then visualised using REVIGO (Supek et al. 2011) and stylised with ggplot (Wickham 2011). An enrichment analysis was then performed between these symbiont conditions using the GO_MWU package, which applies a Mann–Whitney U test to measure whether each GO term is significantly enriched in upregulated or downregulated genes (Wright et al. 2015).

The transcriptomic pipeline used to convert raw reads to transcriptome‐wide visualisation is available in detail on GitHub (https://github.com/TylerJCarrier/AnthopleuraSymbiosis.git) and raw sequence reads have been deposited to the NCBI under the BioProject accession numbers PRJNA1162123 and PRJNA1162900.

### 2.4 Symbiont Phenotype: Holobiont Metabolomics

Approximately 50 mg of tissue was used for a two‐step metabolite extraction using chlorophenylalanine. First, each sample—that was composed of host, microeukaryotic photosymbiont, and other microbes (i.e., a holobiont)—was extracted with 500 μL of methanol:chloroform:water (5:2:2, v:v:v) mixture, which included homogenisation in 1.5 mL screw cap tubes for 3 min using 1 mm inner diameter balls in a Bullet Blender (Net Advance, USA). After centrifugation, 150 μL of supernatant was transferred to a sampling vial for gas chromatography–mass spectrometry (GC–MS) analysis and another 150 μL of supernatant was transferred to a tube for liquid chromatography‐mass spectrometry (LC–MS) analysis. Internal standards were added to each vial. The residual was extracted with 500 μL of methanol and the supernatant was combined into the first step extraction. Each of the 150 μL of supernatant was transferred to the previous vial for GC and the previous tube for LC. All tubes for LC were vacuum dried and suspended in 300 μL of methanol:acetonitrile (5:3, v:v) and analysed by LC coupled with time‐of‐flight mass spectrometry (LC‐TOF‐MS; Agilent Corporation, Santa Clara, CA, USA). All vials for GC were vacuum dried and derivatized with N,O‐Bis(trimethylsilyl)trifluoroacetamide and analysed by GC time‐of‐flight mass spectrometry (GC‐TOFMS; Leco Corporation, St Joseph, USA). Metabolite annotation was performed by comparing the mass spectrum and retention time to using the National Institute of Standards and Technology for GC‐TOFMS, the Human Metabolome Database for LC‐TOFMS (Wishart et al. 2009), or the in‐house library from the Center for Translational Biomedical Research at the University of North Carolina at Greensboro (NC, USA).

The relative abundance of 277 metabolites (all with reference numbers to the Human Metabolome Database) was analysed using MetaboAnalyst (v. 6.0; Pang et al. 2024). The table of metabolite amount was normalised by sum, transformed by Log_10_, and mean‐centered scaled (Table S5). Metabolomic profiles between the three symbiosis phenotypes were compared using a principal coordinates analysis and compared statistically using a permutational multivariate analysis of variance (PERMANOVA) based on 999 permutations within MetaboAnalyst. We then generated a dendrogram to display the relatedness between all samples. Pairwise comparisons by fold change analysis were then performed between *B. muscatinei* or *E. marina* and aposymbiotic sea anemones. The comparison with *E. marina* was also used to determine which metabolites are differentially abundant in aposymbiotic individuals because this symbiotic phenotype is most metabolically similar; hence, this was the most conservative assessment. We then generated a heatmap of the differentially abundant metabolites. Lastly, we used the differentially abundant metabolites in each of the three symbiotic phenotypes to determine which biological processes (based on KEGG; Kanehisa and Goto 2000) are enriched for *A. elegantissima* that is in symbiosis with either *B. muscatinei* or *E. marina* .

### 2.5 Symbiont Phenotypes: Bacterial Community

Total DNA was extracted from all tentacles and the body column for each symbiotic phenotype using the GeneJet Genomic DNA Purification Kit (Thermo Scientific). DNA was quantified using a Qubit Fluorometer (Life Technologies) and diluted to 5 ng/μL using RNase/DNase‐free water. Bacterial sequences were then amplified using primers for the V3/V4 regions of the 16S rRNA gene (Klindworth et al. 2013). Products were purified using the Axygen AxyPrep Mag PCR Clean‐up Kit (Axygen Scientific), indexed using the Nextera XT Index Kit V2 (Illumina Inc.), and then purified again. At each clean‐up step, fluorometric quantitation was performed using a Qubit, and libraries were validated using a Bioanalyzer High Sensitivity DNA chip (Agilent Technologies). Illumina MiSeq sequencing (V3, 2 × 300 bp paired‐end reads) was performed in the Department of Bioinformatics and Genomics at the University of North Carolina at Charlotte (NC, USA).

Raw reads and quality information were imported into QIIME 2 (v. 2022.11; Bolyen et al. 2019), where forward and reverse sequences were paired using VSEARCH (Rognes et al. 2016), filtered by quality score, and denoised using Deblur (Amir et al. 2017). QIIME 2‐generated ‘features’ were analysed as amplicon sequence variants (ASVs; Callahan et al. 2017) and were assigned taxonomy using SILVA (v. 138; Quast et al. 2013). Sequences matching to chloroplasts, mitochondria, and Archaea were discarded. These steps filtered our dataset from 407,620 raw reads to 282,844 high‐quality reads (mean: 7857 reads; range: 1,001–16,762 reads). The filtered table was rarified to 1,000 sequences (Figure S3).

Four measures of alpha diversity (total ASVs, Faith's phylogenetic distance, McIntosh evenness, and McIntosh dominance) were calculated for all samples. These values were compared between host species using a one‐way analysis of variance (ANOVA) in Prism (v. 9.0.0) and were followed by a Tukey's post hoc test for pairwise comparisons. Taxonomy of these communities was then summarised for each sample and pooled by host species. Unweighted and weighted UniFrac (Lozupone and Knight 2005) distances were calculated in QIIME 2, a principal coordinate analysis was performed in QIIME 2, visualised in Prism, and stylised in Adobe Illustrator (v. 24.0.1). Permutational analysis of variance (PERMANOVA) and permutational multivariate analysis of dispersion (PERMDISP), along with their respective pairwise comparisons, were performed within QIIME 2 to test whether community composition and dispersion varied amongst the three symbiotic phenotypes. Lastly, we compared the relative abundance of the primary bacterial taxa (i.e., a *Sphingomonas* spp. ASV) between symbiotic phenotypes using a one‐way ANOVA.

We then downloaded all *Sphingomonas* ASVs (V4 region of the 16S rRNA gene) that are known to associate with cnidarians, as systematically reviewed by McCauley et al. (2023). These 258 cnidarian ASVs as well as the single *A. elegantissima* ASV were aligned and a maximum likelihood phylogeny was constructed using the IQ‐TREE platform (Trifinopoulos et al. 2016). This phylogeny was then visualised using the Interactive Tree of Life (Letunic and Bork 2021) platform and stylised using Adobe Illustrator.

Our QIIME‐based pipeline used to convert raw reads to visualisation is presented in detail on GitHub (https://github.com/TylerJCarrier/AnthopleuraSymbiosis.git) and raw sequence reads have been deposited to the NCBI under the BioProject accession numbers PRJNA1162123 and PRJNA1162900.

## 3 Results

### 3.1 Reference Genome

**FIGURE 2:**
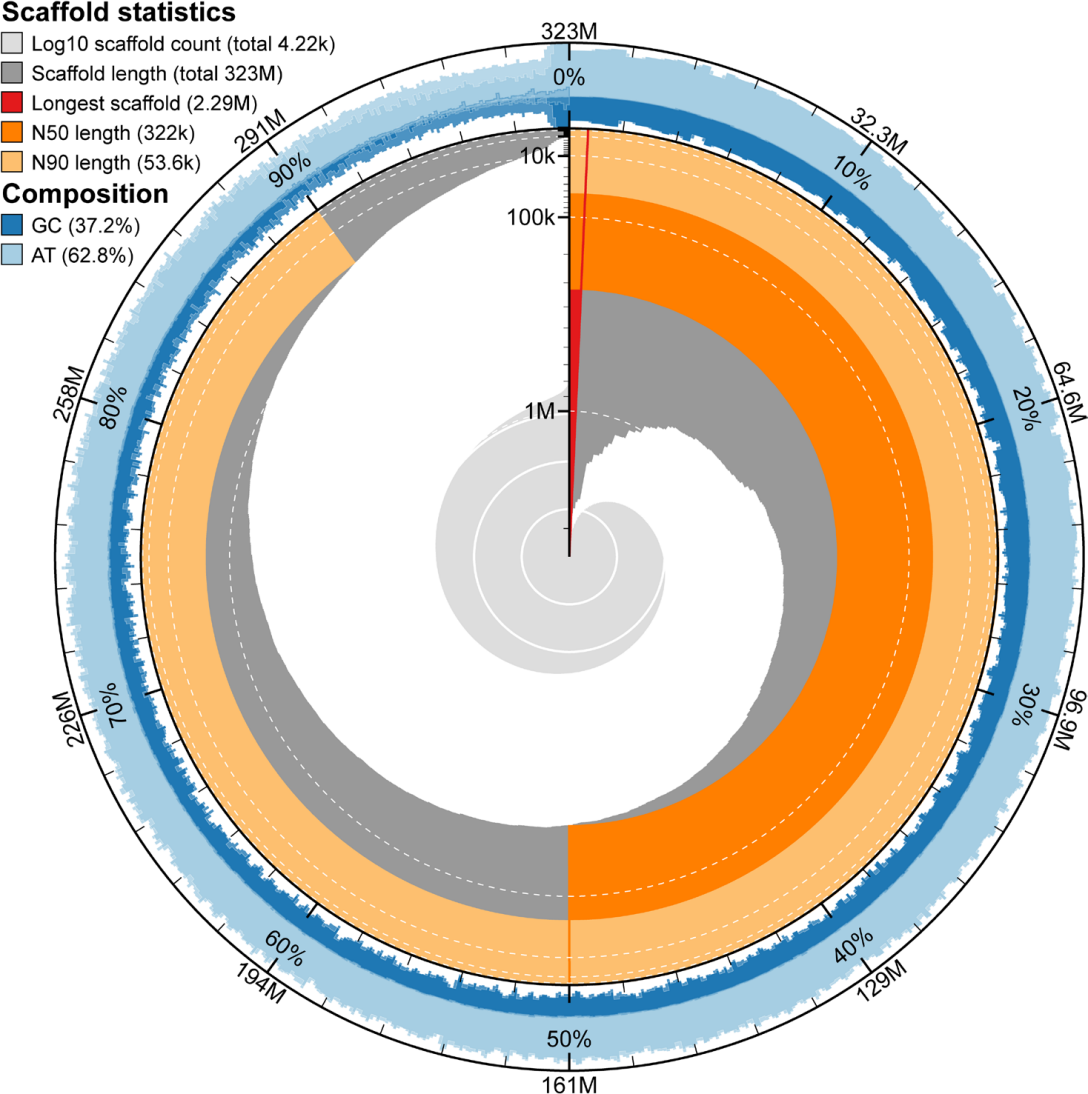
Assembly statistics for the genome of the sea anemone *Anthopleura elegantissima* . The main plot is divided into size‐ordered bins around the circumference of this 323 Mb assembly. Sequence length distribution is shown in dark grey, with the plot radius scaled to the longest sequence present in the assembly (2.29 Mb, as shown in red). The orange and pale‐orange arcs show the N50 and N90 sequence lengths [i.e., the sequence length of the shortest contig at 50% (322 Kb) and 90% (53.6 Kb) of the total assembly length]. The pale grey spiral shows the cumulative sequence count on a log_10_ scale, with the white scale lines showing successive orders of magnitude. The blue areas around the outside of the plot show the distribution of GC (dark blue) and AT (light blue) percentages in the same bins as the inner plot.

Heterozygous frequencies amongst individual sea anemones ranged from 0.003 to 0.005 (Figure S1; Table S1), with the most homozygous individual originally collected from Clatsop Spit (OR, USA). The 322 Mb reference genome produced from this individual had 4,217 scaffolds that totalled ~323 Mb, with 50% and 90% of the genome length in scaffolds being 322 and 53.6 kb or larger, respectively (Figure 2; Table S2). Approximately 0.3% of the scaffolds that were previously in the polished genome matched the eukaryotic photosymbionts. Moreover, ~72.2% (16,191 of 22,420) of all predicted proteins in the *A. elegantissima* reference genome could be annotated (i.e., assigned GO and/or InterPro identifications; Table S3).

### 3.2 Symbiont Quantification

As shown previously by Secord and Muller‐Parker (2005), *A. elegantissima* in symbiosis has orders of magnitude more symbionts (i.e., ~21.3× to 40.0×) than aposymbiotic individuals (one‐way ANOVA, *p* < 0.0001; Tukey's post hoc, *p* < 0.0001) and that sea anemones in symbiosis have a similar number of symbionts to each other (Tukey's post hoc, *p* = 0.503) (Figure S2).

### 3.3 Symbiont‐Mediated Shift in the Host Transcriptome

**FIGURE 3:**
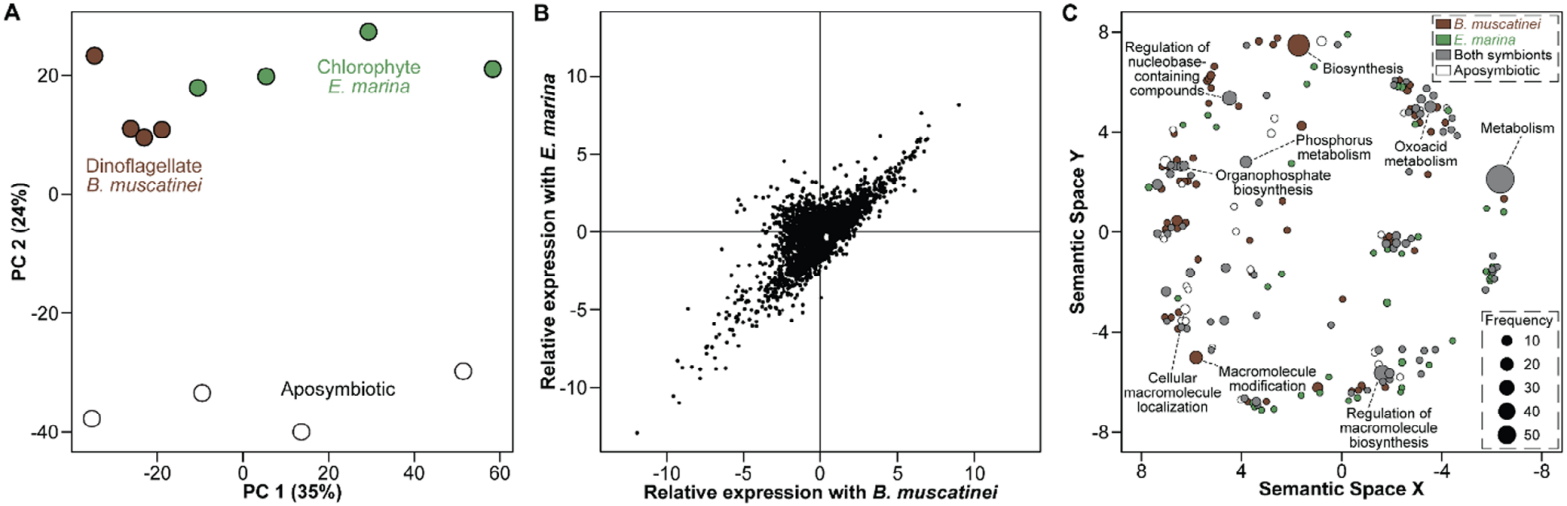
Distinct gene expression profiles across symbiotic phenotypes. (A) The transcriptome of the sea anemone *Anthopleura elegantissima* differed significantly between those harbouring the dinoflagellate *Breviolum muscatinei* (brown), the chlorophyte *Elliptochloris marina* (green), or were aposymbiotic (white). (B) There were ~4,200 differentially expressed genes across these symbiont conditions. The majority of these were up‐regulated when associating with either of the photosymbionts relative to aposymbiosis (top right), up‐regulated when aposymbiotic relative to associating with either of the photosymbionts (bottom left), up‐regulated when associating with *B. muscatinei* and down‐regulated when associating with *E. marina* relative to aposymbiosis (bottom right), or up‐regulated when associating with *E. marina* and down‐regulated when associating with *B. muscatinei* relative to aposymbiosis. (C) Major gene ontology groups for sea anemones that were in symbiosis with *B. muscatinei*, *E. marina* , both symbionts (grey), or were aposymbiotic.

Gene expression of *A. elegantissima* differed significantly between each symbiosis phenotype (ANOSIM, R: 0.49, *p* < 0.001), with individuals harbouring either symbiont being more similar to each other than to aposymbiotic sea anemones (Figure 3A). Symbiosis attributed to the differential expression of 18.9% of the 22,420 genes that were predicted in the *A. elegantissima* genome (Figures S4, S4–S5; Table S7). The largest proportion of differentially expressed genes was shared between sea anemones that were or were not (i.e., aposymbiotic) associated with a photosymbiont (Figure 3B, S5 and Table S7). A smaller proportion of differentially expressed genes was unique to harbouring *B. muscatinei* or *E. marina* (Figure 2B, S5; Table S7). There were 837 differentially expressed genes that exhibited a two‐fold change in expression between sea anemones that hosted either symbiont relative to those that were aposymbiotic, with 41% and 59% of these being up‐and down‐regulated, respectively (Figure S6). Of the up‐regulated genes with a two‐fold change in expression, 121 genes were unique to sea anemones hosting *B. muscatinei*, 98 genes were unique to sea anemones hosting *E. marina* , and 127 genes were up‐regulated when hosting either symbiont (Figure S6). Of the down regulated genes, 48 genes were unique to sea anemones hosting *B. muscatinei*, 232 genes were unique to sea anemones hosting *E. marina* , and 211 genes were down‐regulated when hosting either symbiont (Figure S6).

Differentially expressed genes in aposymbiotic *A. elegantissima* relative to hosting either symbiont are primarily related to the biosynthesis of diverse metabolic products (e.g., amino acid, glucose, and organophosphates). This main gene category was complemented by cellular macromolecule localization, epithelial fluid transport, ligase activity, lipid hydroxylation, and ribonucleotide binding (Figure 3C, S7, S8, Table S8). Sea anemones that associated with either photosymbiont, on the other hand, upregulated genes relating to different, diverse metabolic products, the regulation of respiration (due to macromolecule biosynthesis and nucleobase‐containing metabolism), circadian rhythm proliferation, host–microbe interaction, immunity, neurogenesis, and reproduction and development (Figure 3C, S7, S8, Table S8).

Within sea anemones that are associated with either photosymbiont, those associating with the dinoflagellate *B. muscatinei* predominantly upregulated genes related to nucleotide biosynthesis (~80% by relative frequency), while also having higher expression of genes for organic acid transmembrane transport, phosphorelay signal transduction, and cell wall biogenesis (Figure 3C, S8–S10, Table S8). *A. elegantissima* in symbiosis with the chlorophyte *E. marina* had a smaller number of upregulated genes. These genes had functions related to the regulation of cellular size, polyol biosynthesis, detection of chemical stimuli, branched‐chain amino acid transport, and reproduction and development (Figure 3C, S8–S11, Table S8). Only a few functions relating to carbon and nitrogen metabolism were significantly enriched relative to all differentially expressed genes for sea anemones in symbiosis with the dinoflagellate *B. muscatinei* (Figure S12). Lastly, 10 genes were differentially expressed when associating with either of the photosymbionts relative to aposymbiosis that had inverse expression patterns. All 10 of these differentially expressed genes with inverse expression patterns were upregulated when associating with *E. marina* and predominantly pertained to reproduction and development (e.g., a Homeobox protein), the regulation of organismal processes, and the response to peptide hormones (Figure S10).

### 3.4 Symbiont‐Mediated Shift in Holobiont Metabolome

**FIGURE 4:**
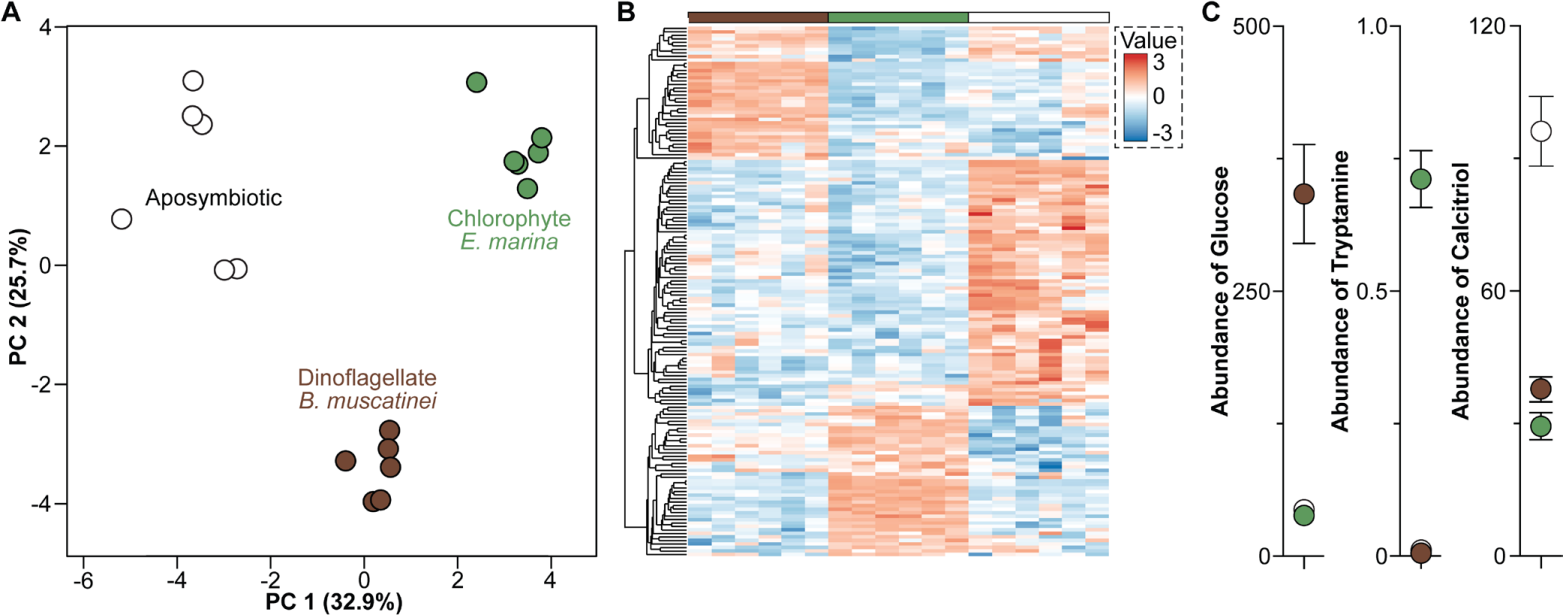
Distinct metabolic profiles across symbiotic phenotypes. (A) The metabolome of the sea anemone *Anthopleura elegantissima* holobiont differed significantly between individuals in symbiosis with the dinoflagellate *Breviolum muscatinei* (brown), the chlorophyte *Elliptochloris marina* (green), or were aposymbiotic (white). (B) Of all the differentially abundant metabolites, 21.9% and 28.5% of these were at a significantly higher amount in sea anemones that were in symbiosis with *B. muscatinei* or *E. marina* , respectfully, while 49.6% of these differentially abundant metabolites were at a significantly higher amount in aposymbiotic individuals. (C) Examples of metabolites that were at a significantly higher amount in for individuals in symbiosis with *B. muscatinei* (left) or *E. marina* (center) included glucose and tryptamine, while calcitriol was higher aposymbiotic sea anemones.

The metabolic profiles of the *A. elegantissima* holobiont differed significantly between each symbiotic phenotype (PERMANOVA, F: 102.4, *R* ^2^: 0.932, *p* < 0.001), with individuals harbouring either symbiont being more similar to each other than to aposymbiotic sea anemones (Figure 4A, S13). More than half (54.5%) of the metabolites were differentially abundant in either of these symbiotic phenotypes (one‐way ANOVA, *p* < 0.05). Nearly half of those metabolites (49.6%) were over‐abundant in aposymbiotic individuals, while approximately a quarter were over‐abundant in those harbouring either *B. muscatinei* (21.9%) or *E. marina* (28.5%). Differentially abundant metabolites in aposymbiotic sea anemones were, for example, involved in the biosynthesis of amino acids [e.g., isoleucine (*p* = 0.046), tyrosine (*p* = 0.007), and tryptophan (*p* = 0.019)], steroids [e.g., calcitriol, (*p* < 0.001); Figure 4B,C], and lipids [e.g., glycerylphosphorylethanolamine (*p* = 0.005)]. Sea anemones in symbiosis with *B. muscatinei* were enriched with metabolites involved in the biosynthesis of glycerol and sugars [e.g., glucose (*p* < 0.001) and oxalacetic acid (*p* < 0.001)]. Lastly, individuals in symbiosis with *E. marina* were enriched with metabolites involved in the biosynthesis of amino acids [e.g., glutamic acid (*p* = 0.005), tryptamine (*p* < 0.001), and tyramine (*p* < 0.001); Figure 4B,C] and energy production [i.e., the TCA cycle; e.g., citric acid (*p* = 0.009) and malic acid (*p* < 0.001)].

### 3.5 Low‐Diversity Bacterial Community

**FIGURE 5:**
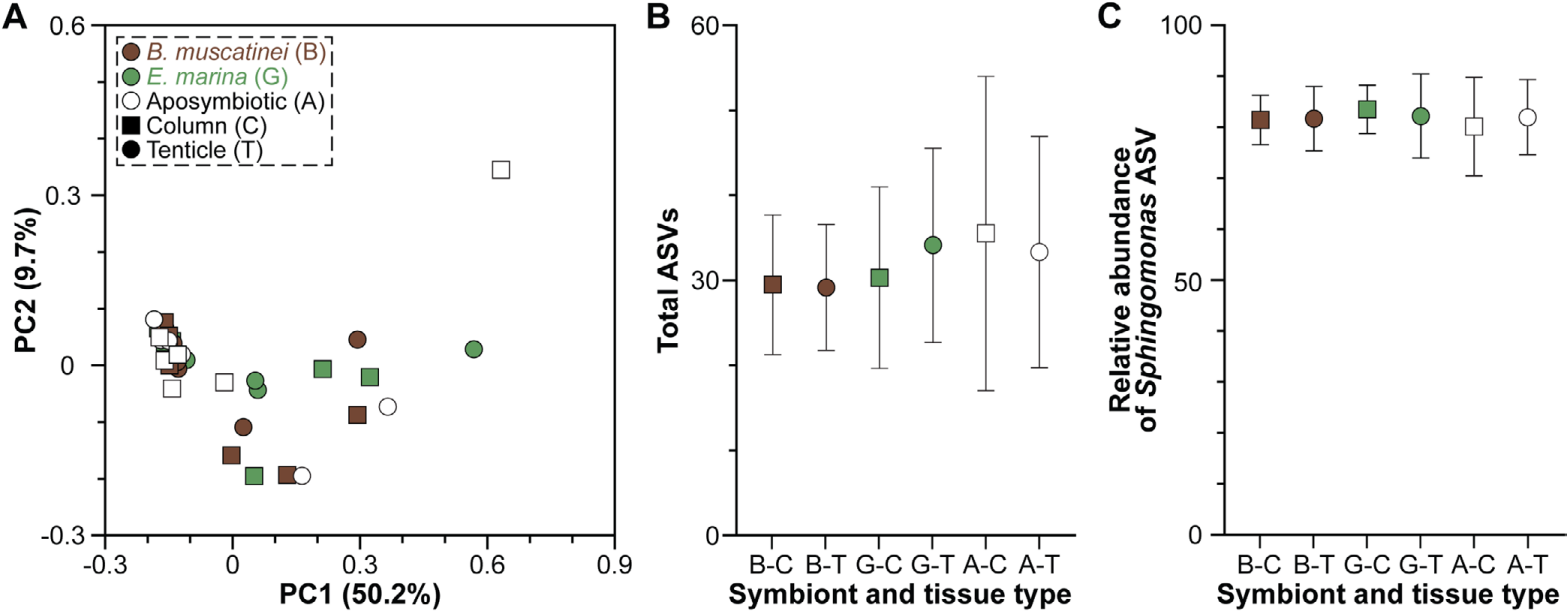
Stable bacterial community across symbiotic phenotypes. The bacterial community associated with the sea anemone *Anthopleura elegantissima* did not differ in community composition (weighted UniFrac) (A) or in the number of bacterial taxa (i.e., ASVs) that were observed (B) between the three symbiont phenotype [the dinoflagellate *Breviolum muscatinei* in brown (B), the chlorophyte *Elliptochloris marina* in green (G), and aposymbiotic in white (A)] or tissue type [squares and circle repesent column (C) and tentacle (T), respectively]. This bacterial community was primary dominated by a single *Sphingomonas* ASV, the relative abundance of which did not differ between symbiotic phenotype or tissue type (C). Data for B and C are average ± standard error (*n* = 6).

The membership and composition of the bacterial community associated with *A. elegantissima* was similar across each of the three symbiotic phenotypes (ANOSIM, unweighted UniFrac: *p* = 0.298, weighted UniFrac: *p* = 0.690) (Figure 5A, S14). This bacterial community was also similar in taxonomic and phylogenetic diversity as well as dominance and evenness (*p* = 0.999 for each) (Figure 5A, S15). This high degree of similarity in alpha and beta diversity was because the bacterial community associated with *A. elegantissima* was primarily composed of a single, uncharacterised *Sphingomonas* (α‐proteobacteria) ASV (81.9%; Figure S15). The relative abundance of this *Sphingomonas* ASV was also similar between these three symbiotic phenotypes (one‐way ANOVA, *p* = 0.352; Figure S15). Moreover, this ASV was closely related to the diverse *Sphingomonas* taxa previously identified to associate with a phylogenetically diverse group of anthozoan cnidarians (e.g., Actinarians and Scleractinians; Figure S16; McCauley et al. 2023). Other members of the bacterial community associated with *A. elegantissima* included γ‐proteobacteria (4.8%), Bacteroidia (3.2%), SAR324 (1.0%), and Actinobacteria (0.6%) (Figure S17).

## 4 Discussion

Animals often rely on microbial symbionts for diverse nutrients and metabolic products that supplement their limited biosynthetic capability (McFall‐Ngai et al. 2013; Zilber‐Rosenberg and Rosenberg 2008). Nutritional symbionts of animals, for example, provide essential amino acids or reduce sulphur compounds that serve as electron donors to fix carbon dioxide autotrophically (Douglas 1998; Dubilier et al. 2008). Corals engage in a similar metabolic associations with the photosynthetic unicellular eukaryotes that provide them with the majority of their energy (Davy et al. 2012; Rosset et al. 2021). Efforts to decode the molecular dialogue have expanded in recent years to other cnidarian species, with a particular emphasis on the sea anemone *E. diaphana* (Baumgarten et al. 2015; Jacobovitz et al. 2023; Roberty et al. 2024). We find that the sea anemone *A. elegantissima* in each symbiotic phenotype has distinct transcriptional and metabolic, but not bacterial, signatures under laboratory conditions. Moreover, we find that *A. elegantissima* is dominated by a single *Sphingomonas* species, suggesting that this sea anemone could potentially have a functionally relevant prokaryotic symbiosis across these phenotypes.

Signatures of symbiosis for *A. elegantissima* tended to follow one of three patterns. The first, and most pronounced, related to being in a general state of symbiosis. This was primarily observed at the transcriptional level, where the majority of differentially expressed genes (~70.5%) across diverse biological processes were between aposymbiotic sea anemones and those that hosted either of the photosynthetic symbionts. A symbiosis‐related transcriptional pattern is widely observed throughout animals, including sponges (Marulanda‐Gomez et al. 2024), worms (Pees et al. 2024), and squid (Kremer et al. 2013). Moreover, this pattern is also observed in the sea anemones *A. elegantissima* —based on a cDNA microarray of the holobiont (Rodriguez‐Lanetty et al. 2006)—and *E. diaphana* (Lehnert et al. 2014) as well as the temperate coral *Astrangia poculata* (Changsut et al. 2022). The processes related to being in symbiosis are also commonly observed, which include metabolism and respiration (Turnbaugh et al. 2008), circadian rhythm (Nyholm and McFall‐Ngai 2004), immunity (Thaiss et al. 2016), neurogenesis (Giez et al. 2023), and reproduction and development (Carrier and Bosch 2022). *A. elegantissima* , thus, exhibits a similar organismal‐level response to being in symbiosis that is expected for other cnidarians.

The second symbiosis signature for *A. elegantissima* relates to the specificity of associating with either of the two photosymbionts. Sea anemones harbouring the dinoflagellate differentially expressed genes predominantly relating to nucleotide biosynthesis amongst other GO terms, while those associating with the chlorophyte had a more even expression of many functional categories (e.g., cell size regulation, reproduction, and vitamin and amino acid transport). Processes identified from these differentially expressed genes translated to the metabolite profiles, as the metabolite profiles for holobionts containing *B. muscatinei* were glycerol and sugar based, while *A. elegantissima* associating with *E. marina* was primarily based on amino acids. These metabolic differences between the symbionts directly contribute to physiological and life history differences between sea anemones in each symbiotic phenotype (Minnick 1984; Trench 1971a, 1971b). Sea anemones hosting *E. marina* tend to reproduce sexually, while being in symbiosis with the more productive *B. muscatinei* promotes cloning by fission (Bergschneider and Muller‐Parker 2008; Bingham et al. 2014). Consistent with this is that *A. elegantissima* that were in symbiosis with *E. marina* differentially expressed genes relating to reproduction and development, while sea anemones with *B. muscatinei* did not.

The third signature for *A. elegantissima* was the lack of a symbiotic influence, which specifically related to the bacterial community associated with this sea anemone. A stable bacterial community dominated by a single *Sphingomonas* taxon was unexpected for three reasons. First, a major change in host physiological state commonly results in a compositional shift in the associated microbial community (Carrier and Reitzel 2017; Kohl and Carey 2016). Second, microbiome diversity spans from a single obligate symbiont to a diverse, mostly facultative community (O'Brien et al. 2019). Sea anemones and other cnidarians are commonly observed to associate with a diverse microbiome composed of 100–1000 s of microbial species (Bourne et al. 2016; McCauley et al. 2023). Third, it has been previously shown that *A. elegantissima* and *E. marina* both associate with a symbiont‐specific microbiome (Morelan et al. 2019; Röthig et al. 2016). Transferring animals into stable laboratory conditions commonly leads to compositional shifts and a reduction in the diversity of the host‐associated bacterial community (Carrier and Reitzel 2017; Kohl et al. 2014). Our mesocosm conditions were provided directly and independently with ambient seawater and, thus, this should have maintained a symbiont‐specific microbiome signature similar to what was reported for *A. elegantissima* that was sampled directly from the field (Morelan et al. 2019).

An alternate explanation of a potential laboratory influence on the bacterial community is that this *A. elegantissima* population predominantly associates with a single *Sphingomonas* taxon. An animal species that associates with a primary microbial taxon that is otherwise expected to interact with a diverse microbiome has been observed [e.g., the sponge *Halichondria panicea* (Knobloch et al. 2019)]. *Sphingomonas* is a commonly observed genus in the bacterial community of cnidarians, suspected to potentially cause a band disease in the elliptical star coral *Dichocoenia stokesi* , and can be a DNA extraction kit contaminant (McCauley et al. 2023; Morelan et al. 2019; Richardson et al. 1998; Salter et al. 2014). The latter is a possibility because this study did not include this type of control. If *Sphingomonas* were removed, then the bacterial community of *A. elegantissima* from near‐ambient mesocosms would have been nearly depleted of detectable bacterial taxa; thus, contamination seems unlikely. The functional role of *Sphingomonas* or a more diverse microbial community remains uncertain across each symbiotic phenotype for the sea anemone *A. elegantissima* .

Collectively, this study has provided a reference genome for *A. elegantissima* as well as assessed whether this sea anemone has a unique transcriptional, metabolomic, and bacterial signature when in symbiosis with the dinoflagellate *B. muscatinei*, the chlorophyte *E. marina* , or when aposymbiotic. Providing this series of resources has set the framework to compare the *A. elegantissima* genome to other sea anemones, corals, and cnidarians (Zimmermann et al. 2023) to determine the host‐microbe dialogue during symbiont shuffling (e.g., in response to light; Cunning et al. 2015), how these partnerships break down in response to future climate conditions (Brown 1997), and to perform experimental infections with thermally resilient photosymbionts (Herrera et al. 2021). *A. elegantissima* is, thus, another tractable experimental cnidarian system that continues to provide insights into a symbiosis that is critically important for both temperate and tropical ecosystems.

## Author Contributions

J.L.D. and B.L.B. collected all samples. J.L.D. performed symbiont counts. H.E. and T.J.C. generated and annotated the reference genome. T.J.C. and J.M. performed the transcriptomic analysis. T.J.C. and A.M.R. performed the metabolic analyses. T.J.C. performed the microbiome analysis. T.J.C. drafted the manuscript, and all co‐authors provided critical feedback.

## Conflicts of Interest

The authors declare no conflicts of interest.

## Supporting information


Figures S1‐S17.

Tables S1‐S8.

## Data Availability

All data from this manuscript been deposited to the NCBI under the BioProject accession numbers PRJNA1162123 and PRJNA1162900. Bioinformatic pipelines used to process these data types are available on GitHub (https://github.com/TylerJCarrier/AnthopleuraSymbiosis.git).
