# Supplementary material for: Symbiont‐Mediated Metabolic Shift in the Sea Anemone Anthopleura elegantissima
Source: Mol Ecol. 2025 Mar 17;34(8):e17722. doi: 10.1111/mec.17722 (PMC11974494; doi:10.1111/mec.17722)
Supplement: Supplementary file 1 — Figures S1‐S17. [file MEC-34-e17722-s001.pdf]

Supplementary Information for:

Symbiont-mediated metabolic shift in the sea anemone *Anthopleura elegantissima*

Tyler J. Carrier[1,2], Holland Elder[3,4], Jason Macrander[5], James L. Dimond[6], Brian L. Bingham[6,7], & Adam M. Reitzel[1,2*]

[1] Department of Biological Sciences, University of North Carolina at Charlotte, Charlotte, USA

[2] Center for Computational Intelligence to Predict Health and Environmental Risks, University of North Carolina at Charlotte, Charlotte, USA

[3] Department of Integrative Biology, Oregon State University, USA

[4] Australian Institute of Marine Science, Townsville, Australia

[5] Biology Department, Florida Southern College, Lakeland, USA

[6] Shannon Point Marine Center, Western Washington University, Anacortes, USA

[7] Department of Environmental Sciences, Western Washington University, Bellingham, USA

[*] Corresponding author: adam.reitzel@charlotte.edu

This PDF file includes:

Figures S1 to S17

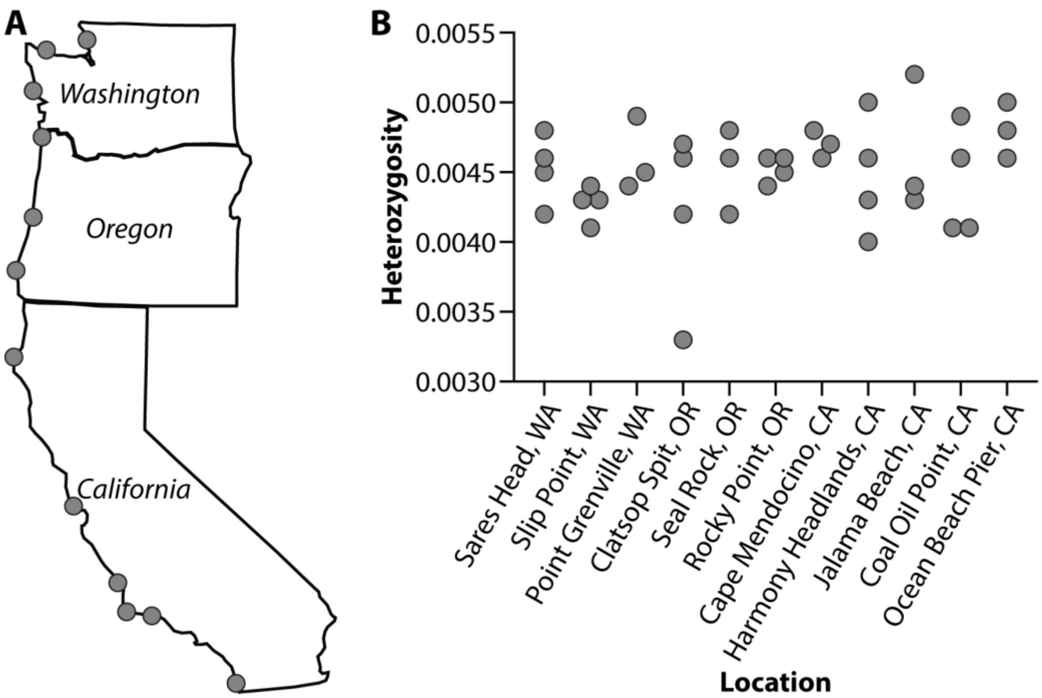

Figure S1: Map of sampling sites and homozygosity. Map of the west coast of the United States marked with the geographic location of the sampling sites where the sea anemone *Anthopleura elegantissima* was collected (A) to identify the most homozygous individual (B) to generate a reference genome. Locations are organized from north to south.

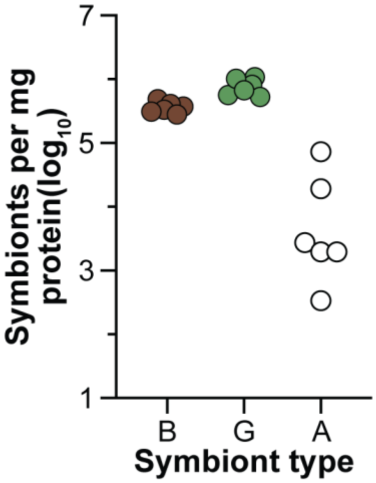

Figure S2: Symbiont abundance. Symbiont abundance (per mg of protein) in individuals of the sea anemone *Anthopleura elegantissima* that were later used to profile the transcriptome, metabolome, and microbiome. B (brown) and G (green) denotes individuals in symbiosis with the dinoflagellate *Breviolum muscatinei* (formally *Symbiodinium*) and the chlorophyte *Elliptochloris marina*, while A represented aposymbiotic individuals.

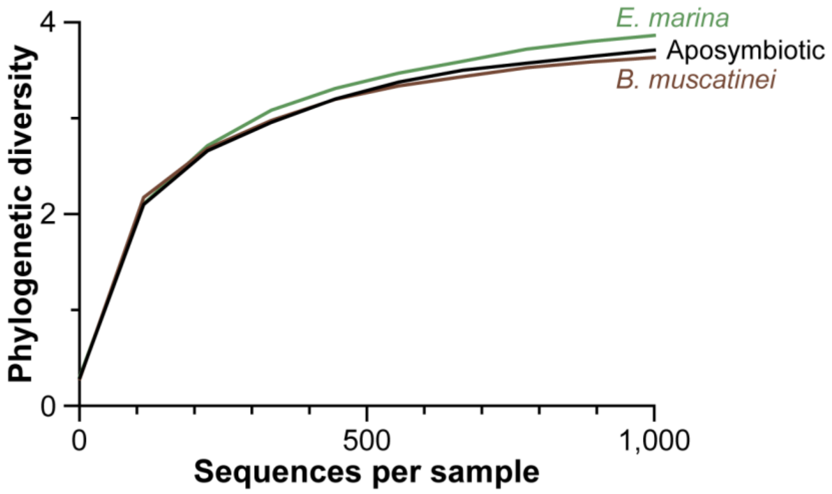

Figure S3: Rarefaction for bacterial community analysis. Alpha rarefaction curve for the bacterial community associated with the sea anemone *Anthopleura elegantissima* that were in symbiosis with the dinoflagellate *Breviolum muscatinei* (brown), the chlorophyte *Elliptochloris marina* (green), or aposymbiotic individuals (black). The rarefaction depth was 1,000 sequences per sample.

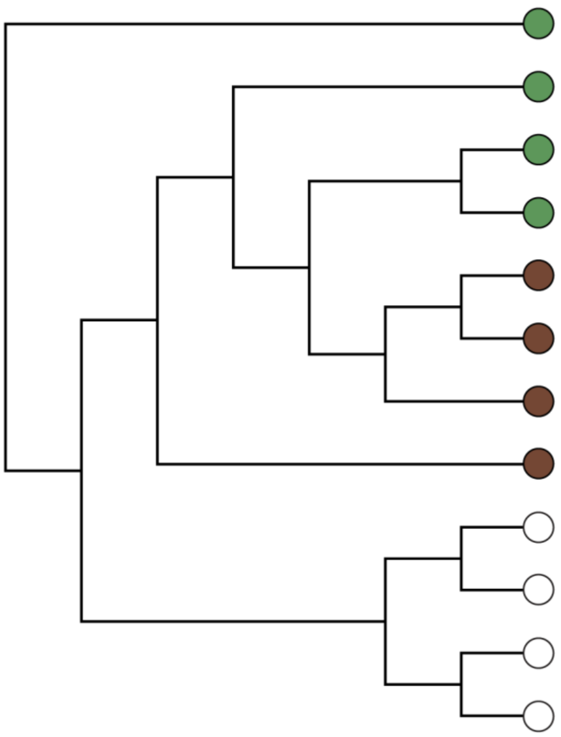

Figure S4: Dendrograms of the transcriptomes across symbiont states. Similarity of the transcriptome of the sea anemone *Anthopleura elegantissima* grouped based on the individual were in symbiosis with the dinoflagellate *Breviolum muscatinei* (brown), the chlorophyte *Elliptochloris marina* (green), or were aposymbiotic (white).

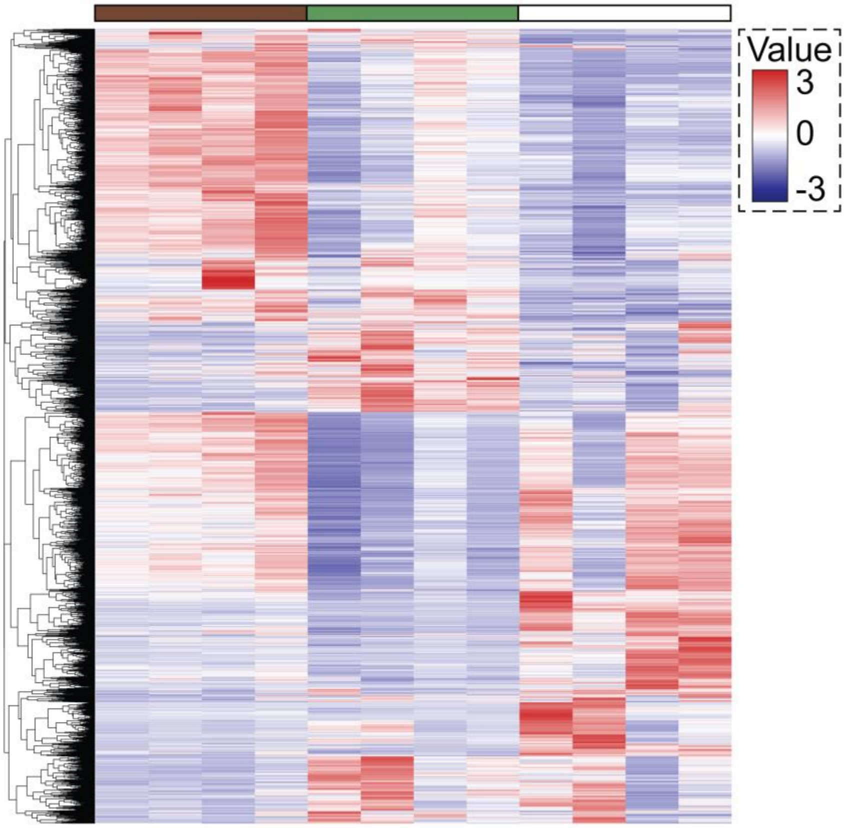

Figure S5: Heat map of all differentially expressed genes. Comparative expression levels of the ~4,200 differentially expressed genes for sea anemone *Anthopleura elegantissima* that were in symbiosis with the dinoflagellate *Breviolum muscatinei* (brown), the chlorophyte *Elliptochloris marina* (green), or were aposymbiotic (white). Scale represents $\log_2$ fold change.

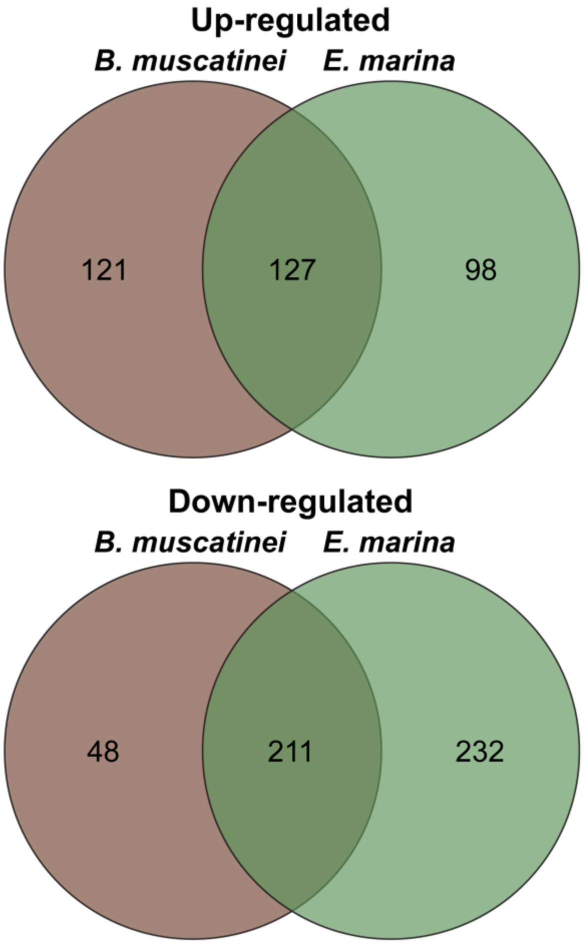

Figure S6: Venn diagram of differentially expressed genes that exhibited a two-fold difference in expression. Numeric representation of differentially expressed genes that exhibited a two-fold difference in expression and that were up (top) or down (bottom) regulated between sea anemones hosting the dinoflagellate *Breviolum muscatinei* (brown) or the chlorophyte *Elliptochloris marina* (green) relative to those that were aposymbiotic.

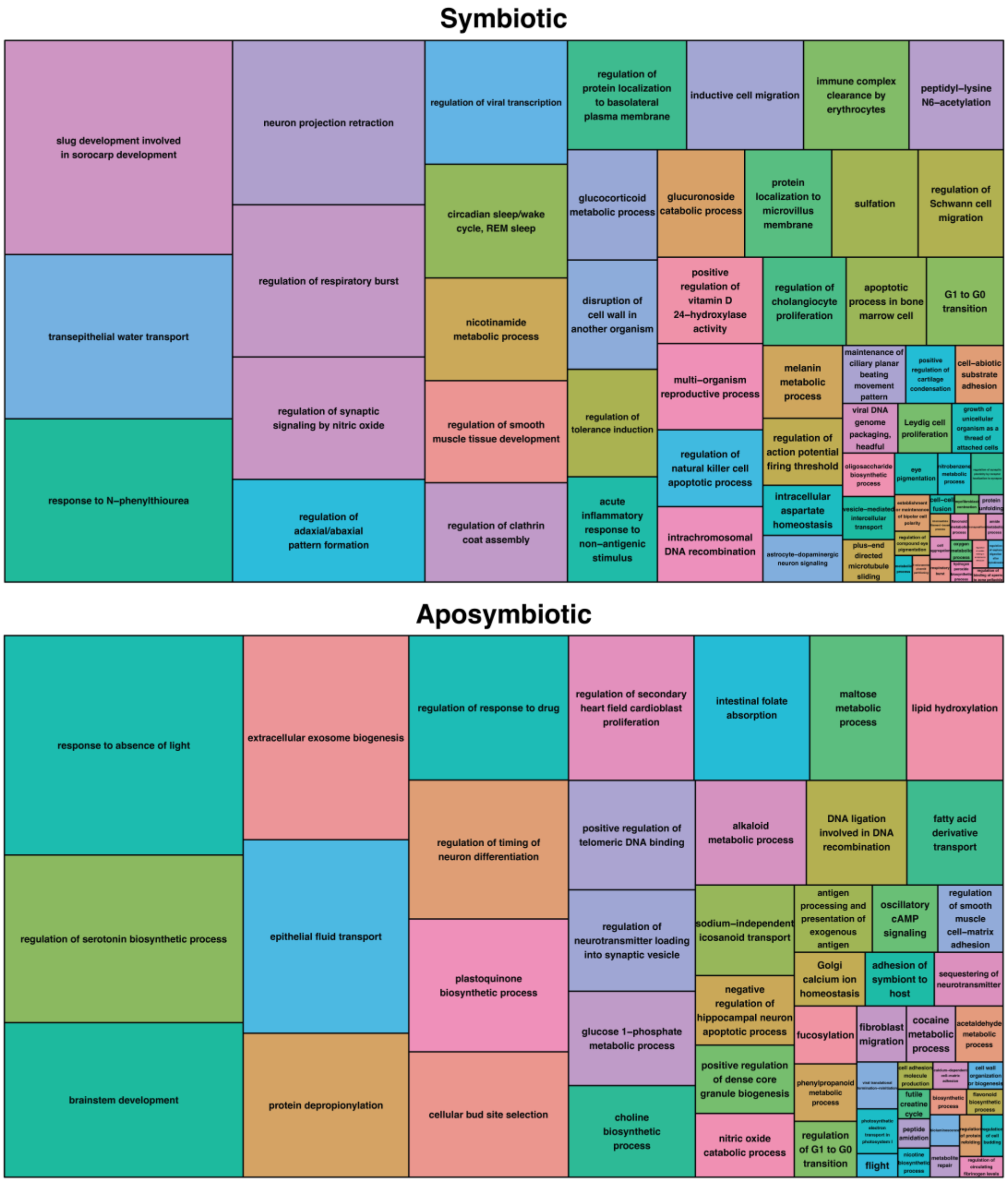

Figure S7: Biological processes unique to associating with symbiosis. TreeMaps (via REVIGO) of the representative terms that are unique to being symbiotic (top) or aposymbiotic (bottom). Size of each box is relative to each symbiont.

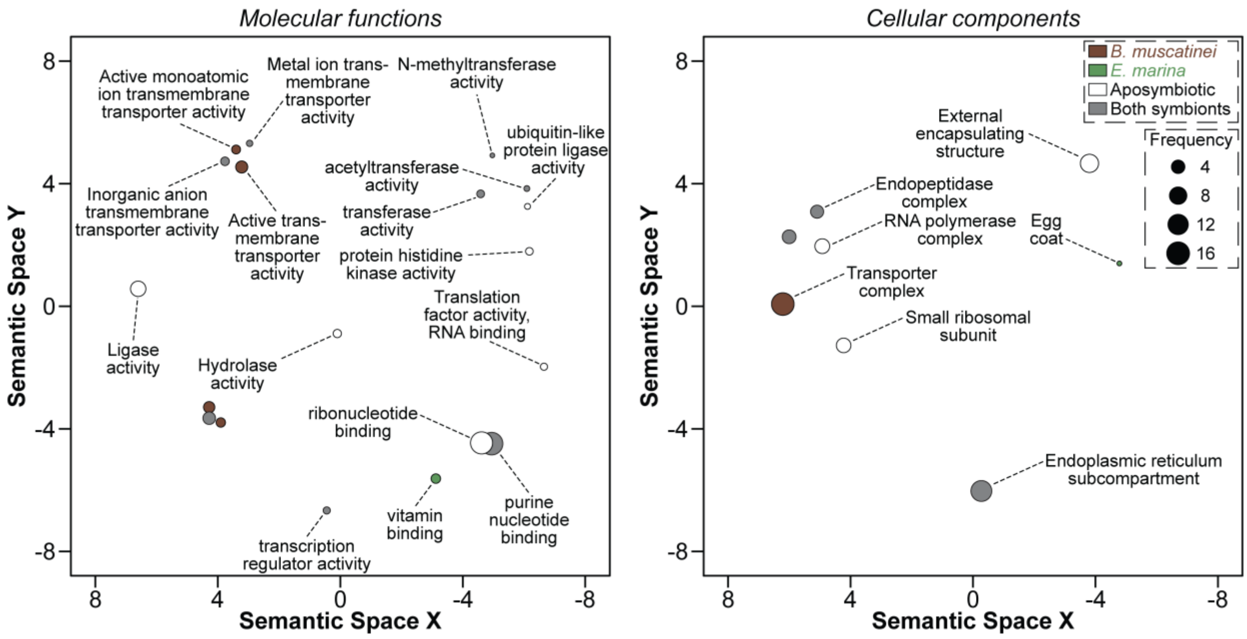

Figure S8: Major gene ontology groups across symbiont states. Frequency of molecular function (left) and cellular components (right) for the sea anemone *Anthopleura elegantissima* that were in symbiosis with the dinoflagellate *Breviolum muscatinei* (brown), the chlorophyte *Elliptochloris marina* (green), both symbionts (gray), or aposymbiotic (white).

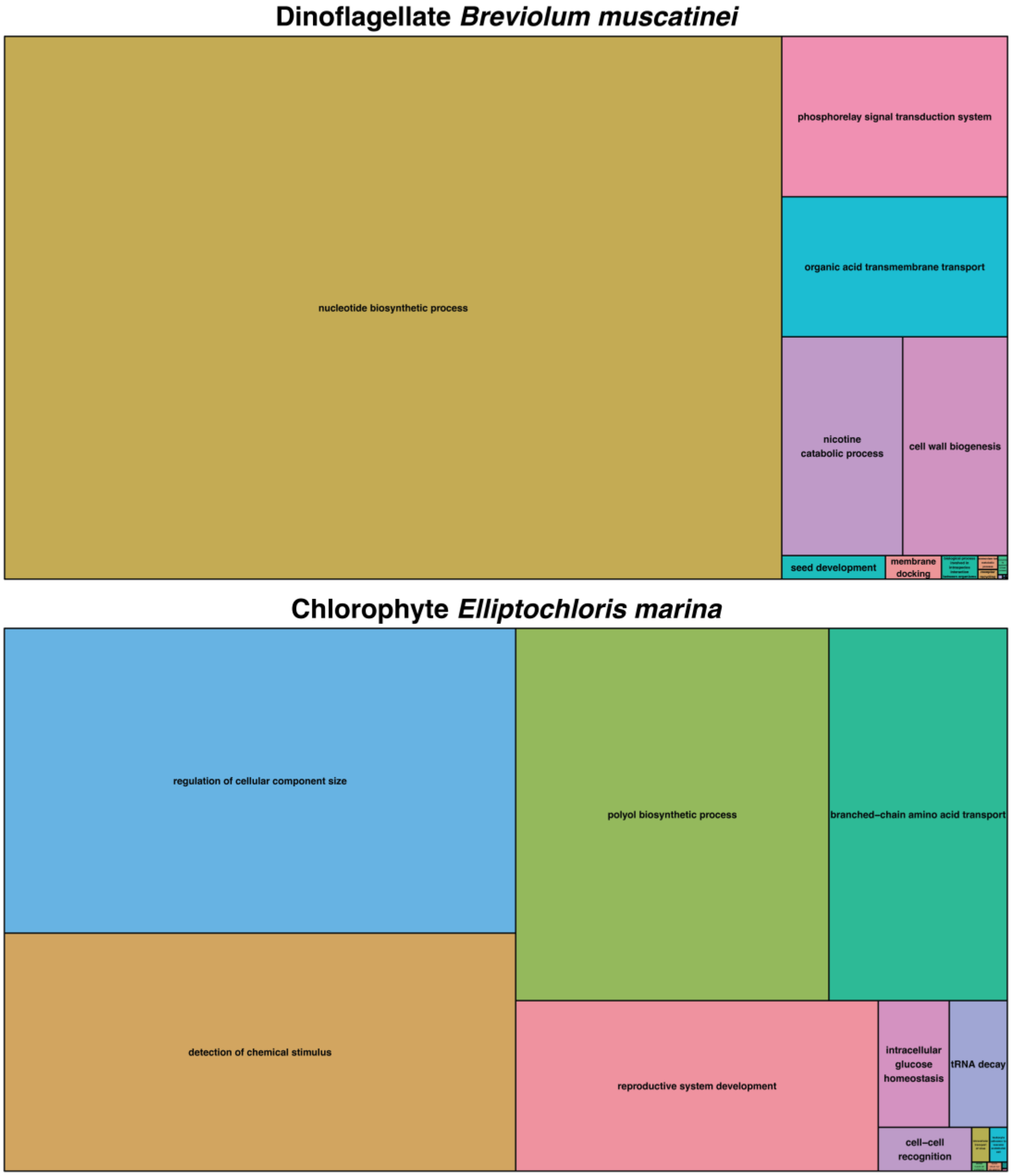

Figure S9: Biological processes unique to associating with either symbiont. TreeMaps (via REVIGO) of the representative terms and the biological processes within each that are unique to associating with either the dinoflagellate *Breviolum muscatinei* (top) or chlorophyte *Elliptochloris marina* (bottom). Size of each box is relative to each symbiont.

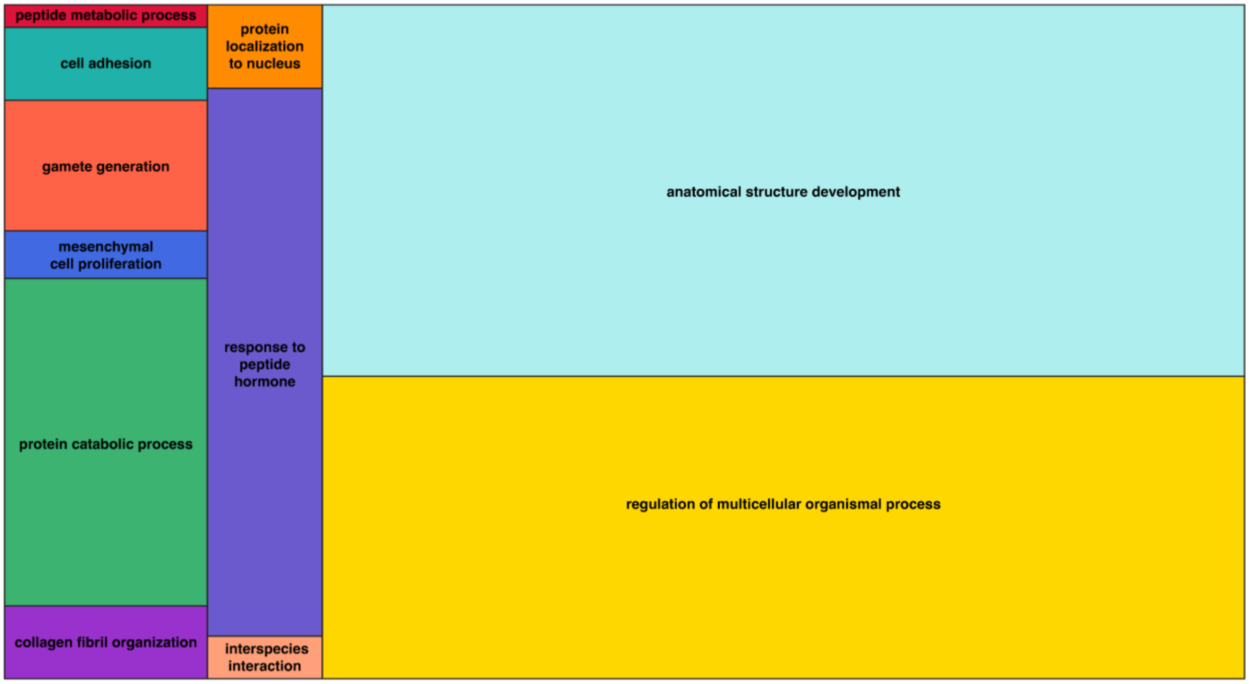

Figure S10: Biological processes that exhibit inverse expression patterns between symbionts. TreeMaps (via REVIGO) of the representative terms that are uniquely upregulated in one symbiont and downregulated in the other relative to being aposymbiotic. All ten of the differentially expressed genes in this category were upregulated when associated with the chlorophyte *Elliptochloris marina* and downregulated when associated with the dinoflagellate *Breviolum muscatinei*.

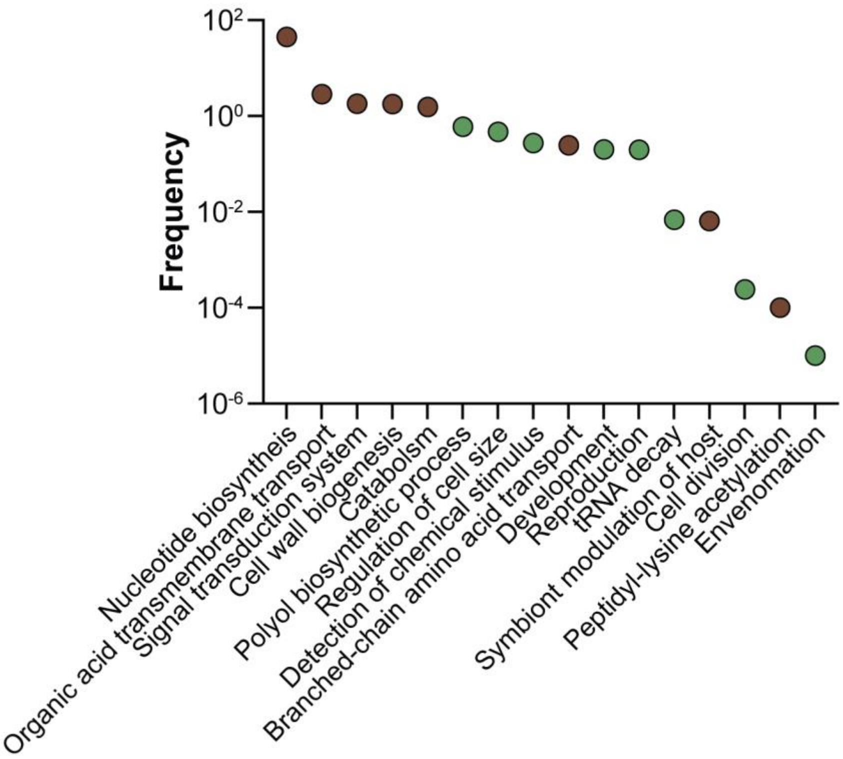

Figure S11: Frequency of representative biological processes unique to associating with either symbiont. Absolute values from the TreeMaps (via REVIGO) of the representative terms unique to associating with either the dinoflagellate *Breviolum muscatinei* (top) or chlorophyte *Elliptochloris marina* (bottom). Size of each box is relative to each symbiont.

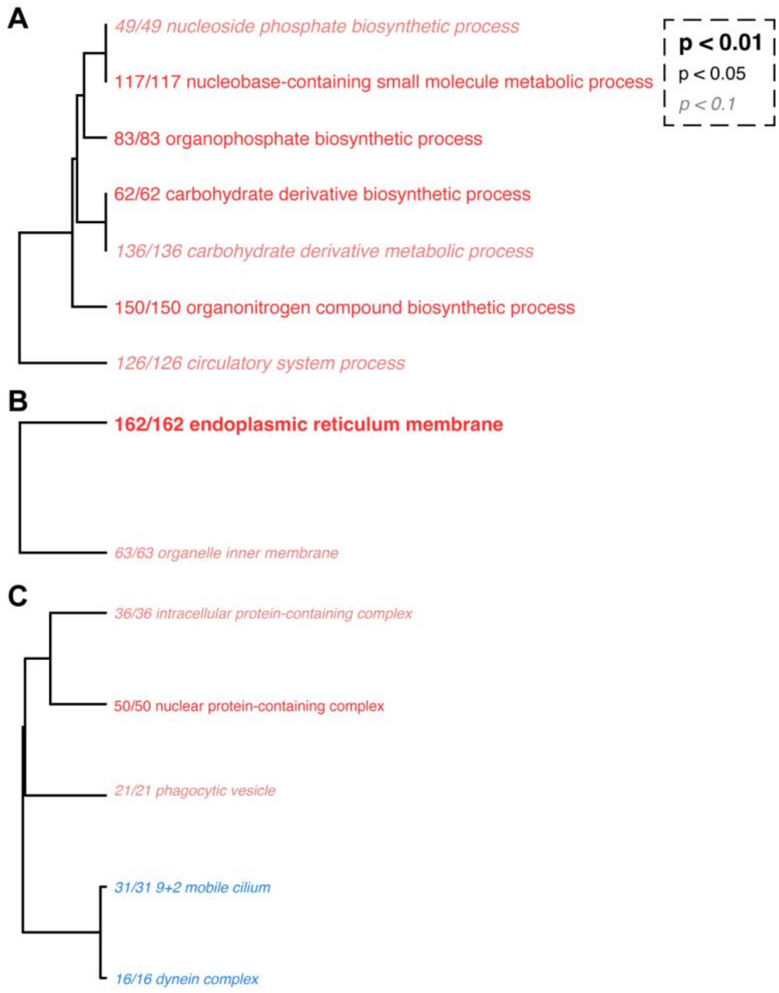

Figure S12: Enriched gene ontology groups. Several gene ontology groups were over (red) or under (blue) enriched between the sea anemone *Anthopleura elegantissima* harbored the dinoflagellate *Breviolum muscatinei* (A: biological processes; B: cellular components) compared to aposymbiotic individuals as well as between those associated with *B. muscatinei* and the chlorophyte *Elliptochloris marina* (C: cellular components).

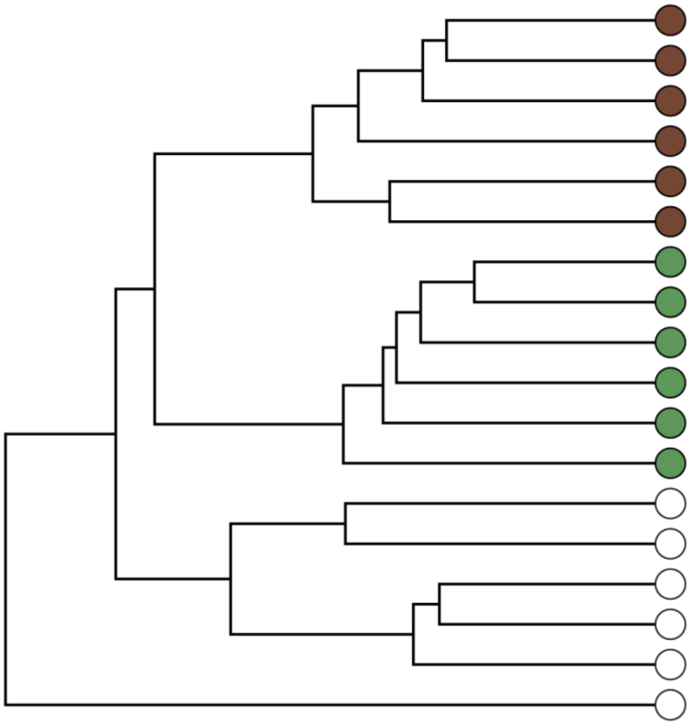

Figure S13: Dendrograms of metabolomes across symbiont states. Similarity of the metabolome of the sea anemone *Anthopleura elegantissima* grouped based on the individual were in symbiosis with the dinoflagellate *Breviolum muscatinei* (brown), the chlorophyte *Elliptochloris marina* (green), or were aposymbiotic (white).

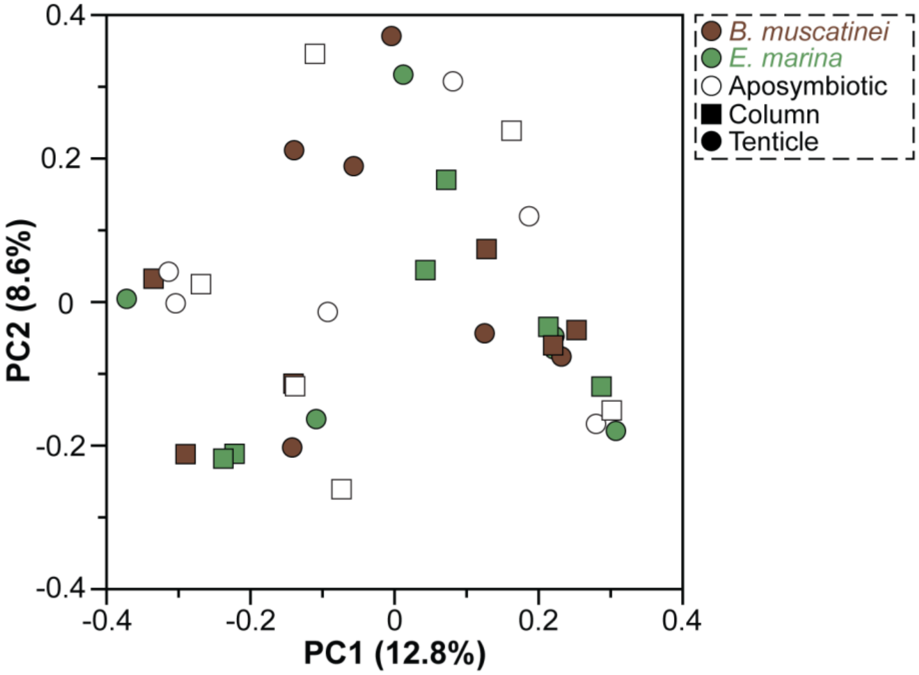

Figure S14: Similar membership in the bacterial community across symbiont states. The bacterial community associated with the sea anemone *Anthopleura elegantissima* did not differ in community membership (unweighted UniFrac) community between the three symbiont states (the dinoflagellate *Breviolum muscatinei* in brown, the chlorophyte *Elliptochloris marina* in green, and aposymbiotic in white) or tissue type (squares and circle repesent column and tenticle, respectively).

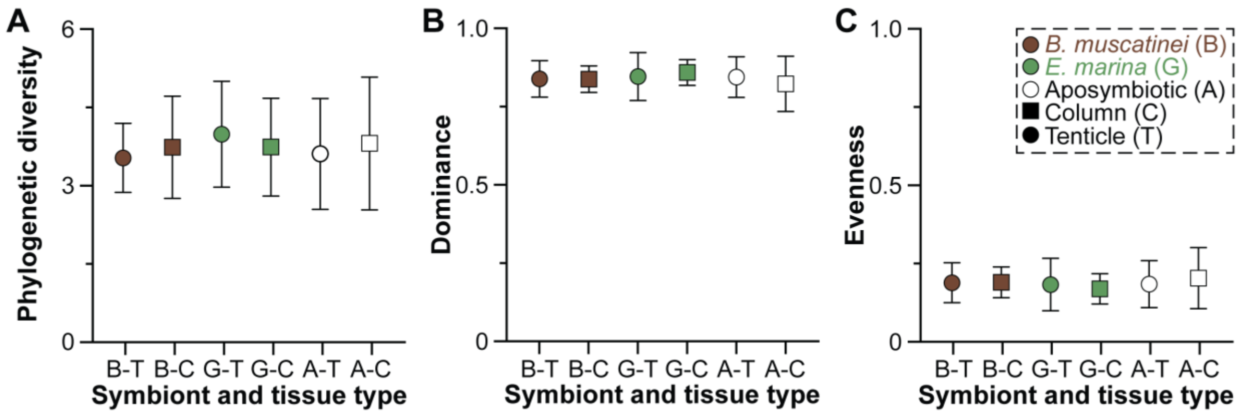

Figure S15: Consistent diversity in the bacterial community across symbiont states. The bacterial community associated with the sea anemone *Anthopleura elegantissima* did not differ in the diversity (A), dominance (B), or evenness (C) of their bacterial community between the three symbiont states (the dinoflagellate *Breviolum muscatinei* in brown, the chlorophyte *Elliptochloris marina* in green, and aposymbiotic in white) or tissue type (squares and circle repesent column and tenticle, respectively). All values are average ± standard error.

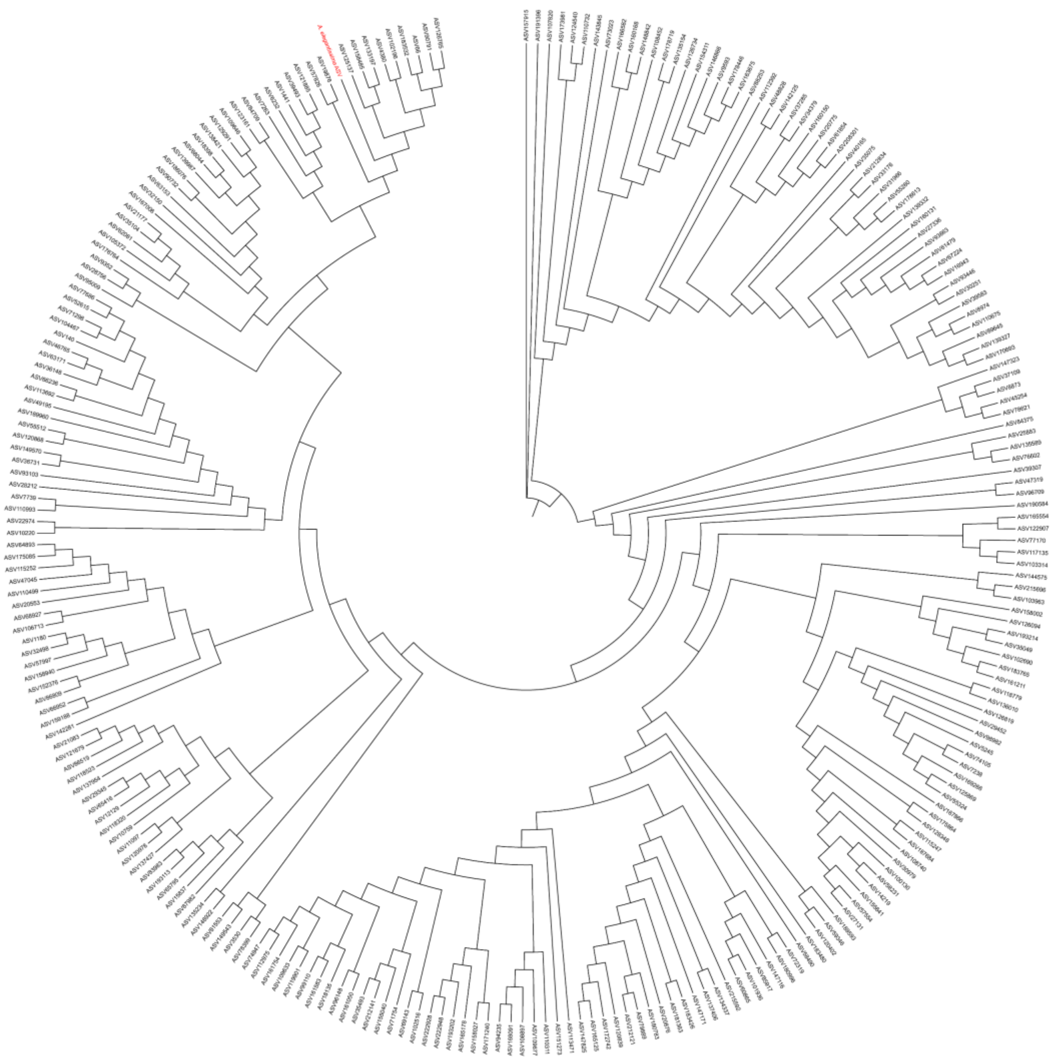

Figure S16: *Sphingomonas* phylogeny. Phylogeny of the *Sphingomonas* amplicon sequencing variants (ASV) that are consistently found in the bacterial communities of cnidarians [with names maintained from McCauley *et al.* (2023)] and their relationship to the *Sphingomonas* found to dominate the bacterial community of the sea anemone *Anthopleura elegantissima*.

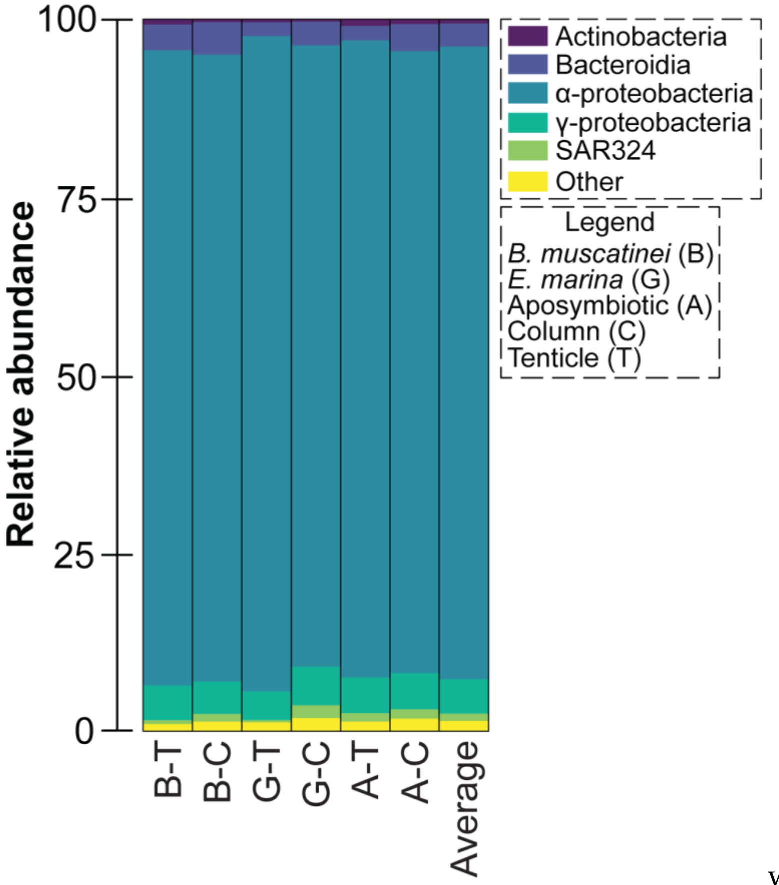

w

Figure S17: Bacterial profiles across symbiont states. Phylum-level profiles of the bacterial communities associated with the sea anemone *Anthopleura elegantissima* that were in symbiosis with the dinoflagellate *Breviolum muscatinei* (B), the chlorophyte *Elliptochloris marina* (G), or were aposymbiotic (A). Tissues samples of tentacles (T) and body column (C) were both profiled.
